# Harnessing the power of neuroplasticity for intervention

**DOI:** 10.3389/fnhum.2014.00377

**Published:** 2014-06-27

**Authors:** Bryan Kolb, Arif Muhammad

**Affiliations:** Canadian Centre for Behavioural Neuroscience, University of LethbridgeLethbridge, AB, Canada

**Keywords:** brain plasticity, neurorehabilitation, recovery of function

## Abstract

A fundamental property of the brain is its capacity to change with a wide variety of experiences, including injury. Although there are spontaneous reparative changes following injury, these changes are rarely sufficient to support significant functional recovery. Research on the basic principles of brain plasticity is leading to new approaches to treating the injured brain. We review factors that affect synaptic organization in the normal brain, evidence of spontaneous neuroplasticity after injury, and the evidence that factors including postinjury experience, pharmacotherapy, and cell-based therapies, can form the basis of rehabilitation strategies after brain injuries early in life and in adulthood.

Over the past 20 years, our understanding of how the brain is changed by experience, usually referred to as *neural plasticity*, has exploded. Once believed to be fairly constant in its organization and function, it has become clear that the brain is inherently capable of changing after injury to enable at least some behavioral restitution. What is less clear, however, is what factors might potentiate (or attenuate) the endogenous response to injury and what rules might guide the reparative changes.

Although the idea of brain plasticity is well recognized in neuroscience and rehabilitation, it is not easily defined and can refer to many changes ranging from behavior to molecular events such as gene expression (e.g., Shaw and McEachern, 2001). Nonetheless, it is possible to identify some general principles that can guide us in harnessing the power of neural plasticity to design new rehabilitation strategies.

## Challenges in studying neural plasticity and behavior

### Correlation is not causation

By its nature, behavioral neuroscience is correlational. Consider an example. If a laboratory animal is trained in some type of motor task such as reaching for food with fine forelimb movements, we can look for accompanying changes in the structure of cells in the putative motor circuit (e.g., Withers and Greenough, 1989). Now if we administer a drug such as nicotine while animals learn the task we may find enhanced learning and a correspondingly larger change in the neural circuit (e.g., Gonzalez et al., 2006). But what caused what? The drug may have acted directly on the motor circuit making it easier to change, or the drug could have had some less direct effect such as increasing activity, which in turn enhanced the behavior and synaptic changes. Furthermore, just because neurons change with the learning does not mean that those changes have anything to do with the memory of the task *per se*, but could simply reflect the motor activity inherent in performing the task. As a result, behavioral studies seeking to link behavioral and synaptic changes are often criticized for “simply showing correlates.” The problem is even larger when we are studying the effects of treatments on motor recovery after brain injury. Stroke may produce motor deficits and these deficits may be reduced with a treatment such as nicotine and this may be correlated with synaptic changes. There is little doubt that the drug changed behavior but we do not know what caused the synaptic changes.

One solution is to try to break the correlations and see if the behavioral change persists. For example, if we can prevent the structural changes but the behavior still improves, then they are not related. But even if we cannot break the correlation, we still do not have proof of causation. The challenge is to prove how the synaptic changes arose, such as by showing that a change in gene expression is caused by a treatment and that the epigenetic change leads to the behavioral changes. For the majority of studies, this is impractical. Proving causation in behavioral neuroscience is an extremely difficult process but for the purposes of designing treatments to facilitate improvement from brain injury, it probably does not matter. As a beginning it is sufficient to demonstrate that behavior is improved and that the improvement is associated with changes in the brain.

### What is recovery?

It is common to refer to functional improvement after brain injury as “recovery.” But this term is ambiguous. It might imply a complete return of function, a marked improvement in function, or just some improvement. It is rare indeed for true return of the pre-injury state to occur. We like to refer to this as the “three-legged cat problem.” If a hindlimb of a cat is seriously injured, it is not uncommon for a veterinarian to amputate the leg. Postoperatively the cat has great difficulty in moving around but over a period of months eventually adapts and can be surprisingly agile. Has the cat recovered? No. True recovery would require the regrowth of the missing limb. Rather, the cat has compensated for the lost limb. It would be simple to design a task to demonstrate things that the cat would have difficulty doing. Nonetheless, for the most part the cat adapts well. A similar phenomenon occurs after brain injury. Functional improvement may occur but there is still dead or dysfunctional tissue. The person has learned to cope with the disability, either physically or cognitively. As we search for mechanisms underlying functional improvement we need to wary of thinking that there is true recovery because this may mislead our investigations (see review by Alaverdashvili and Whishaw, 2013).

### What is the best level of analysis?

Research on the neural basis of rehabilitation can be conducted at many levels of analysis beginning with behavior, and moving increasingly more reductionistic to neural imaging and electrophysiological recording from the scalp, cerebral mapping, invasive physiological recordings, neuronal morphology, genetics and epigenetics, and finally to proteins and other molecules. The choice of level will depend upon the question being asked. For example, if the goal is to develop pharmacological therapies, the appropriate level will likely be more molecular in order to examine synaptic mechanisms or structure. On the other hand, to understand how compensatory neural circuits are organized it makes sense to examine neural circuitry using neural imaging or to identify changes to sensory or motor maps. The key point here is that there is no “correct” or “best” level. Studies need to be at all levels if we are to understand how to design the best therapies.

### What are appropriate animal models?

Although many studies can be undertaken in humans without prior preclinical studies in laboratory animals, our understanding of the mechanisms underlying the neural basis of rehabilitation must usually begin in the laboratory using animal models. The choice of the best animal model is problematic. One significant issue is that larger brains, such as humans, have a relatively larger amount of white matter compared to gray matter than smaller-brained mammals that are normally used in laboratory studies (Zhang and Sejnowski, 2000). One consequence of this is that humans are more likely to have white matter injury than lab animals with similar gray matter injury. Approximately 25% of human strokes involve subcortical white matter injury and although there are several animal models that attempt to mimic human white matter injury, to date none are ideal (Hainsworth and Marjus, 2008; Sozmen et al., 2012). The failure to adequately mimic human white matter injury is problematic in trying to model the neural basis of rehabilitation. Specifically, a real question is whether treatments that are effective in stimulating enhanced compensation in laboratory animals with gray matter injury generalize to people with white matter injury.

A second problem is related to how results are generalized from lab animals to human patients. Animal studies generally have well-defined injuries with far less variance than in human conditions. In addition, animal studies rarely include very large injuries because smaller injuries generally show a much better response to therapies than larger injuries. Human clinical trials typically choose patients with larger injuries, presumably because they are perceived to have greater need of help, even though the proof of principle in a translation to humans might be easier to demonstrate in people with less severe symptoms.

A third problem relates to models outside primary sensory or motor regions. It is common to make unilateral injuries when studying treatments for motor cortex injuries but bilateral injuries when studying cognitive functions such as those performed by the prefrontal cortex, posterior parietal cortex, or medial temporal regions. Bilateral injuries are needed in such studies in rats because unilateral lesions produce only very small deficits. In contrast, humans normally have only unilateral injury to prefrontal, posterior parietal or temporal regions but they can have quite substantial behavioral symptoms. Given that studies of rats with unilateral motor cortex injury have shown that the intact contralateral hemisphere plays a significant role in functional improvement (e.g., Jones and Schallert, 1992; Gonzalez and Kolb, 2003), we might predict a similar mechanism with unilateral prefrontal injuries. Indeed, this may be the reason that the unilateral lesions in rodents show such minimal functional effects. One possible solution here is to focus the animal studies on the neural compensation rather than the behavior, although this seems counterintuitive.

### Brain injury may have effects on somatic organs

One assumption of most studies of rehabilitation and neural compensation is that changes in the nervous system are closely related to functional outcomes. There are a few clinical reports, however, that suggest some relationship between stroke and somatic organ function, and especially kidney function (e.g., Losito et al., 2012). In most such studies the implications are either that cardiovascular problems and renal problems either share predisposing conditions (e.g., high blood pressure) or that renal problems increase the risk of stroke. Another possibility, however, is that brain injury induces functional changes both in brain and somatic organs. A recent study looked at molecular epigenetic changes in kidney, heart, and liver of rats who had suffered ischemic stroke (Kovalchuk et al., 2012). They found changes in all three organs but the effects were largest in the kidney where they found changes in methylation, acetylation, gene expression, and microRNA expression that were still present 4 months poststroke. These changes may significantly alter kidney function, which in turn may influence the course of functional improvement. This study serves as a warning that rehabilitation programs may need to be wary of the effects of stroke on distal tissues and organs.

## General principles of plasticity in normal brain

Before we address treatments that enhance functional outcomes and neural plasticity after brain injury we will briefly review key principles of plasticity in the normal brain.

### Neural plasticity is found in all nervous systems and the principles are conserved

All animals, including very simple ones like *C. elegans*, can show various forms of learning, which is correlated with neuronal plasticity (e.g., Ardiel and Rankin, 2010). This plasticity includes both pre- and postsynaptic changes and are remarkably similar to those observed in animals with much more complex nervous systems. It is believed, for example, that there are NMDA-like changes in learning in both invertebrates and mammals (e.g., Roberts and Glanzman, 2003). There are likely differences in the details, such as the nature of gene expression changes and second messengers but the general principles appear to be conserved across diverse phyla. This is important because it allows researchers to use a wide range of models to search for the neural mechanisms of plasticity.

### A wide variety of experiences alter the brain throughout the lifespan

**Figure 1:**
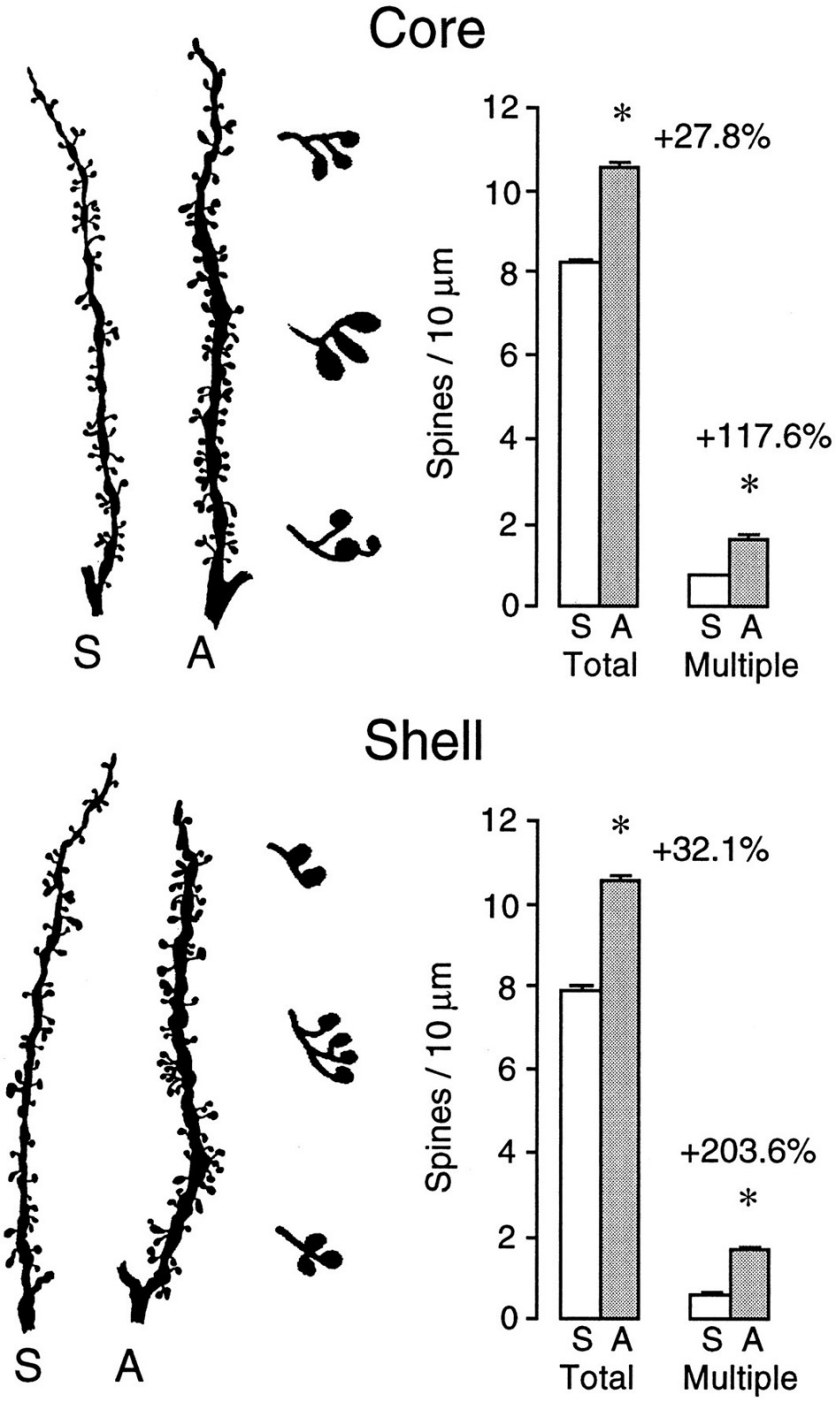
**Camera lucida drawings of representative segments from medium spiny neurons in the core (top) and shell (bottom) subregions of the nucleus of saline- or amphetamine-treated rats**. The drawings to the right of the segments illustrate multiple heading spines, which show a very large drug-induced increase (After Robinson and Kolb, 1997). ^*^p < 0.01.

Virtually any experience can change the brain, especially if there is an associated behavioral change (Table 1). We learn and remember, create new thoughts, and behavior changes throughout our lifetime. Changing behavior requires changes in the neural circuits that underlie it. For example, there are many studies showing that learning neuropsychological tasks is associated with synaptic changes in regions believed to be requisite for the learning (e.g., Greenough and Chang, 1989; Kolb et al., 2008). Similarly, repeated exposure to psychoactive drugs can result in long-lasting changes in behavior, which is correlated with large alterations in morphology of neurons in many brain structures including medial and orbital prefrontal cortex, nucleus accumbens, caudate-putamen, and hippocampus (see Figure 1) (e.g., Robinson and Kolb, 2004). Indeed, it is the capacity of drugs such as amphetamine and nicotine that have led to their use as treatments for brain injury (see below). Indeed, the role of experiences in general in changing the brain is especially important as we search for treatments for brain injuries.

**Table 1:** **Factors affecting the synaptic organization of the normal brain**.

**Factor**	**Example reference**
1. Sensory and motor experience	Greenough and Chang, 1989
2. Task learning	Comeau et al., 2010
3. Gonadal hormones	Mychasiuk et al., 2012
4. Psychoactive drugs	Robinson and Kolb, 2004
5. Neurotrophic factors (e.g., NGF, FGF-2)	Monfils et al., 2008
6. Natural rewards (e.g., sex; social interaction)	Fiorino and Kolb, 2003
7. Prenatal experiences	
7. Social play	Bell et al., 2010
8. Aging	Kramer et al., 2004
8. Stress	McEwen, 2005
9. Anti-inflammatories (e.g., COX-2 inhibitors)	Silasi and Kolb, 2007
10. Diet (e.g., choline)	Meck and Williams, 2003
11. Electrical stimulation:	
Kindling	Teskey et al., 2006
LTP	Monfils et al., 2004
LTD	Monfils and Teskey, 2004
Surface cortical stim	Adkins et al., 2008

### Plastic changes are area dependent

**Figure 2:**
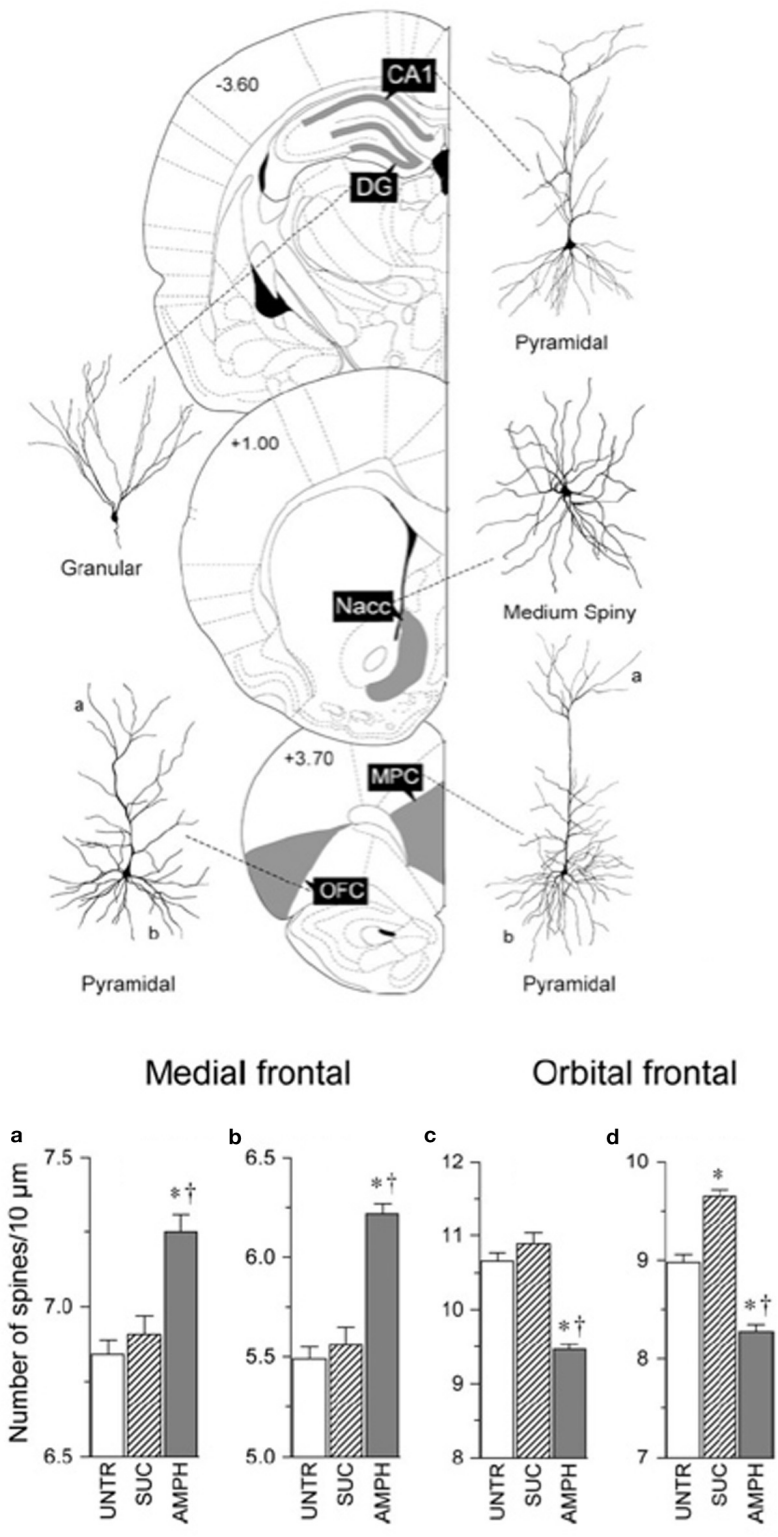
**Top:** Illustration of location of, and cell morphology, for the analysis of the effects of amphetamine on neuronal structure. **Bottom**: Summary of the contrasting effects of amphetamine on the medial frontal and orbital frontal cortex. Abbreviations: UNTR, untrained; SUC, trained to bar press for sucrose; AMPH, trained to bar press for amphetamine (After Crombag et al., 2005). ^*^Differs from control, *p* < 0.05; ^†^differs from sucrose, *p* < 0.05.

It is generally assumed that powerful experiences such as complex housing would produce similar neuronal changes. In fact, it is not uncommon for investigators to choose one structure (often the hippocampus) as a surrogate for how experiences are altering the brain. It has been therefore surprising to us that this is wrong. For example, when one compares the effects of amphetamine on the medial prefrontal and orbital prefrontal cortex, parietal cortex, nucleus accumbens, CA1 of the hippocampus, and the dentate gyrus the effects are wildly different (Crombag et al., 2005). Thus, whereas neurons in the medial prefrontal cortex, nucleus accumbens, and CA1 show increased spine density, neurons in the orbital frontal cortex show a decrease in spine density and the other structures no change at all (see Figure 2). Similarly, when Mychasiuk et al. (2012) examined the effects of prenatal stress on gene expression in hippocampus and medial prefrontal cortex they found over 100 genes changed in each region but there was virtually no overlap in which genes changed. Using one structure or the other (or blood) as a surrogate marker for epigenetic change throughout the brain is clearly misleading.

The area-dependent nature of plastic changes in the brain has obvious implications for developing therapies for brain injury. The therapies need to be informed by where the changes are hoped to occur.

### The degree of plastic change is related to both the relevance of an experience and the intensity or frequency of the events

Experiences that highly relevant to an animal are likely to produce much more rapid neuronal changes than less relevant experiences. Fowl become imprinted on a moving object appearing shortly after their hatching, which in the natural world would normally be a parent. There are immediate changes in the chick's hyperstriatum after visual imprinting including decreased spine density, increased NMDA receptor density, and increased immediate early gene expression (e.g., Horn, 2004). In contrast, experiences that are perceived as irrelevant may not lead to neural changes, even with extensive experience. An example can be seen in the training of pigeons to peck an illuminated key to obtain food vs. training to peck a key to avoid an aversive stimulus. Pigeons come prepared to associate pecking with food but not with aversion avoidance. In the latter case they appear to be unable to learn to avoid shock by pecking. Although we are unaware of any studies searching for plastic changes in the avoidance-type paradigm, it is a safe bet that there would be few if any to be found.

One factor related to relevance is motivation. One reason that laboratory animals are placed on mild food deprivation schedules when they learn tasks is because hungry animals are more highly motivated to solve the tasks to obtain food reward than sated ones. Both listening to and learning music can be a strong motivator for people and provides benefits for stroke recovery (e.g., Sarkamo and Soto, 2012; Grau-Sanchez et al., 2013).

Not only is relevance important, but so is intensity. For example, drug studies show that low doses of psychomotor stimulants produce more restricted synaptic changes than high doses (e.g., Diaz Heijtz et al., 2003). A similar effect can be seen with the duration of complex housing on cortical pyramidal cells (e.g., Greenough and Chang, 1989).

Both these phenomena are directly relevant to designing rehabilitation programs. For example, Kollen et al. (2006) reviewed treatment outcomes after stroke and concluded that the optimal rehabilitation programs encorporate high intensity therapy with a strong emphasis on functional training for relevant tasks. These studies had no measure of brain changes but the behavioral benefits of the therapy provide a strong suggestion that the treatment did alter cerebral organization.

### Plastic changes are time-dependent

Plastic changes can change over time. We noted above that one of the most powerful experiences to change neural networks is exposure to psychomotor stimulants such as amphetamine and nicotine. Cocaine has similar actions: it increases spine density in both medial frontal cortex and nucleus accumbens as effectively as amphetamine (Robinson and Kolb, 1999). After a month of abstinence the effect persists, just as it does for amphetamine and nicotine. However, in contrast to the latter drugs, which show persisting effects after 3 months of abstinence, the effects of cocaine are largely dissipated (Kolb et al., 2003a,b,c), a result consistent with the behavioral and neurophysiological effects of cocaine (Henry and White, 1995). Not only does the effect of cocaine diminish over time, it has recently been shown that it is possible to reverse the effects of cocaine on synaptic function in nucleus accumbens by administering a cystine prodrug, N-acetylcysteine (Moussawi et al., 2011). This result is obviously important for its implications in treating cocaine dependence but more generally in that it shows that neural plastic changes can be reversed.

Time-dependent changes can be seen in many other paradigms as well. Comeau et al. (2010) placed rats in complex environments for varying lengths of time. They then compared the dendritic changes in medial prefrontal and parietal cortex. Previous studies had shown that after 90 days of such housing there were large dendritic changes in parietal cortex but virtually none in prefrontal cortex (Kolb et al., 2003a,b,c). The Comeau et al study found although there was no dendritic change visible after 14 days, there were significant changes after 4 days. The opposite was true in parietal cortex—there were clear changes at 14 days but none at 4 days. This result was unexpected because although it was anticipated that plastic changes might have area-dependent schedules of change, it was not anticipated that one change would replace another. This result has implications for the design of rehabilitation programs. Different brain regions may respond to treatments following a different timetable.

### Experience-dependent changes interact

Although laboratory studies are often designed to present animals with a single large experience (such as a drug), in real life experiences are not singular events. We have experiences beginning *in utero* and continuing throughout life. The effects of these experiences accumulate, a process referred to as metaplasticity (e.g., Abraham, 2009). Basically, metaplasticity reflects a change in the biochemical, physiological, or morphological state of neurons or synapses that alters their subsequent ability to change state. Little is known about these interactions but if we are to understand why brain-injured patients respond differently to treatments, we need to recognize that people have different histories, including drug histories.

Most laboratory studies of metaplasticity have used some type of priming experience such as a drug or hormone followed minutes or days later high frequency electrical brain stimulation to induce long-term potentiation. Another way to study metaplasticity in the laboratory is to provide animals with one significant experience (such as a drug) and then later train animals on behavioral tasks or place them in complex environments. Both experiences are known to produce large changes in cerebral organization but what happens when they are combined? The short story is that previously exposing animals to amphetamine, cocaine, or nicotine (and even prenatal nicotine) dramatically attenuates or blocks the later effect of complex housing or behavioral training (e.g., Kolb et al., 2003a,b,c; Hamilton and Kolb, 2005; Mychasiuk et al., 2014). Similarly, when Muhammad et al. (2011) gave infant rats tactile stimulation with a fine brush 15 min per day from birth until weaning they found an attenuated response to amphetamine in adulthood. They chose tactile stimulation because like drugs and complex housing, tactile stimulation produces large changes in dendritic organization in multiple cerebral regions (e.g., Richards et al., 2012). The found a marked attenuation of the effects of amphetamine given in adulthood. Thus, it appears that drugs can reduce the effects of later experiences but early experiences can also reduce the effects of drugs. This may become important when we try to translate the effects of drugs on recovery in animal studies to humans with a rich lifetime of experiences that may modulate the action of the drugs.

The idea of metaplasticity is likely related to the concept of cognitive reserve, which refers to the differences in cognitive capacity in older people related to a lifetime of intellectual activities (e.g., Barulli and Stern, 2013). For example, Verghese et al. (2003) showed that extensive participation in leisure activities in older people (>75 year) is associated with a reduced risk of dementia. The hypothesis is that cognitive reserves stemming from previous learning experiences play a protective role in coping with neurodegenerative diseases. The idea can be extended to hypothesize that cognitive reserve might also have the benefit of enhancing the efficacy of rehabilitative strategies after brain injury, which would be an example of metaplasticity.

### Plastic changes are age-dependent

The developing brain is more responsive to experiences than the adult brain but there are qualitative differences in response to similar experience at different ages. It is known, for example, that stress in adulthood decreases spine density in medial prefrontal cortex but increases it in orbital frontal cortex (Liston et al., 2006). In contrast, however, prenatal stress produces just the opposite pattern in the adult brain, namely an increase in medial prefrontal cortex and a decrease in orbital frontal cortex (Muhammad et al., 2012). Similar age-dependent qualitative changes can be seen in the effects of complex housing (Kolb et al., 2003c). When adult animals are placed in such environments there is a general increase in spine density in cortical pyramidal cells whereas similar experience at weaning leads to a decrease in spine density. The age-dependent nature of synaptic change is clearly significant when we consider most effective treatments neurological disorders at different ages such as in pediatric vs. adult disorders.

### Both prenatal and preconceptual experience can alter the adult brain

Although the brain continues to develop long after birth, it has become clear in the last decade that prenatal and preconceptual experiences can profoundly alter the adult brain. Exposure to psychoactive drugs, including prescription drugs, stress, and complex housing all change synaptic organization of the prefrontal cortex (e.g., Kolb et al., 2012a,b; Muhammad et al., 2012; Mychasiuk et al., 2013). In addition, mild prenatal stress increases global methylation in prefrontal cortex and hippocampus (Mychasiuk et al., 2011a,b) and alters gene expression in a sexually-dimorphic and and region-specific manner in brains examined at weaning (Mychasiuk et al., 2011a,b). The frontal cortex changes were largely related to neurotransmitter function, whereas hippocampal changes were more prominent in females and concentrated around growth factors. It seems likely that the preconceptual experiences could influence the brain's later response to injury.

Although there have been many reports that parental experiences could influence epigenetics and subsequent health in the offspring (e.g., Barker, 1998; Kaati et al., 2002; Pembrey, 2004), it is only recently that experiences of the parents have been shown to affect epigenetics in the brain of offspring (Mychasiuk et al., 2011a,b, 2013). For example, Mychasiuk et al. (2013) found that paternal stress alters offspring behavior and DNA methylation patterns in a sexually-dimorphic manner, presumably because of experience-dependent effects on spermatogenesis. The authors suggest that brain development is influenced not only by postnatal experiences but is also influenced by earlier maternal and paternal experiences, which combine to produce the various phenotypes and individual differences that we perceive in closely related individuals. Once again, such differences are likely to influence how the brain responds to injury and rehabilitation much later in life.

### Not all plasticity is good

Most studies of brain plasticity and behavior emphasize the idea that plastic changes can support improved cognitive and motor function. But plastic changes are not all good and can interfere with behavior. For example, it is likely that drug-related alterations in prefrontal cortex and other brain regions could underlie some of the maladaptive behavior observed in drug addicts. Another example can be seen in focal hand dystonia in musicians.

Focal hand dystonia refers to abnormal finger and hand positions, cramps, and difficulty in coordinating hand and finger movements. Dystonia can be so disabling that some musicians must give up their occupation. Dystonia is most common in instruments, such as stringed instruments, that require maximal fine finger movements. Although the precise cause of dystonia is still debated, one hypothesis is that it results from extensive practice of the more intensively used hand, which likely results from a distortion of the somatosensory and/or motor maps in the cortex (Candia et al., 2003). Another hypothesis is that the practice leads to abnormal activation patterns in the premotor cortex (Kadota et al., 2010), which in turn could be related to abnormal cortical maps. In either event, the dystonia symptoms appear to result from pathological plasticity.

There are many other examples of pathological plasticity including dementia (Mattson et al., 2001) epilepsy (Teskey, 2001), and pathological pain (Baranauskas, 2001). It is not known if there is pathological plasticity after brain injury but one suggestive example comes from Nudo et al. (1996) who showed that without rehabilitation after injury to the motor cortex, the area regulating hand movements becomes smaller—a phenomenon sometimes referred to as a disuse syndrome. With rehabilitation the hand area retains its cortical representation. The reversal of this type of pathological plasticity is presumably important in understanding the success of restraint-induced therapies.

## Harnassing plasticity for neurorehabilitation

**Figure 3:**
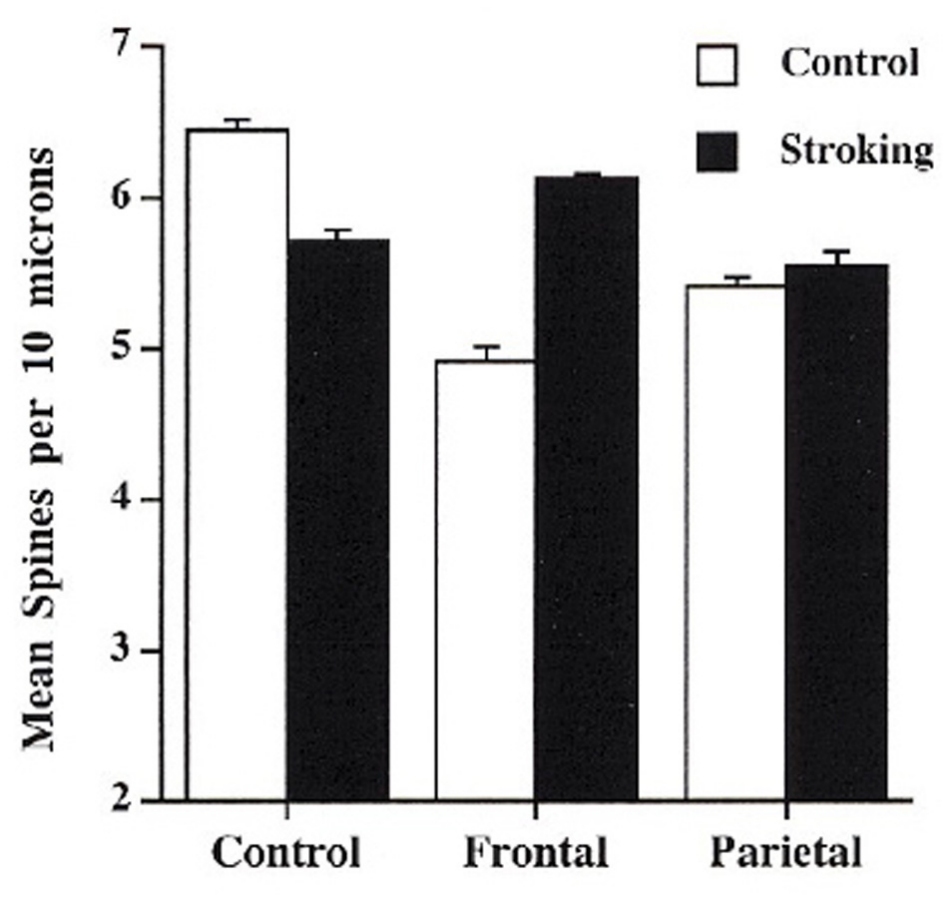
**Effect of tactile stimulation (stroking with a fine brush) on spine density in control, day 3 frontal injury, and day 3 parietal injury in rats (After Kolb and Gibb, 2010)**.

The studies of neural plasticity in normal animals provide a launch pad to develop new strategies for designing rehabilitation programs (for an extensive review, see Cramer et al., 2011). Our basic assumption is that experiences that change the normal brain will likely produce similar, and hopefully even larger, changes in the injured brain. This is often the case but not always. For example we have shown that tactile stimulation in early development has a profound effect on neural organization and behavior (e.g., Richards et al., 2012) so we anticipated that tactile stimulation might be a good treatment for brain injury, which it is (see Richards et al., 2012, below). What we had not expected, however, was that the synaptic changes in normal and brain-injured animals would be qualitatively different and that different injuries would respond with quite different changes. Thus, whereas there was a *decrease* in spine density in cortical pyramidal neurons in sham operates, which was correlated with enhanced motor and cognitive skills, there was an *increase* in animals with perinatal prefrontal injuries (see Figure 3). Nevertheless, the latter animals showed remarkable functional recovery in both cognitive and motor behaviors (e.g., Kolb and Gibb, 2010). Curiously, animals with posterior parietal injuries showed no effect of the treatment on spine density: they had an increase in dendritic length instead (not shown), which was also correlated with functional improvement. These results illustrate our ignorance of brain plasticity and behavior. In some ways we might think of brain injury followed by a treatment as a form of metaplasticity. The injury stimulates spontaneous reparative changes that then interact with our treatment.

As we consider the factors summarized in Table 1, and how they might be used to design rehabilitation treatments, we can group them into four somewhat arbitrary categories: postinjury experience, pharmacotherapy, cell-based therapy, and diet. Our focus here will be on studies of laboratory animals and mostly will use synaptic changes inferred from Golgi studies as a measure of brain plasticity.

### Behavioral experience

There is a rich history of treatments on physiotherapy for skeletomuscular dysfunctions so it is reasonable to design treatments for brain injury based upon such treatments. For example, patients could practice impaired behaviors with the goal of relearning or improving functions. Historically, most such therapies have been based upon hunch and habit rather than RCTs or preclinical studies. The success of such studies is difficult to quantify but a large international meta-analysis by Teasell et al. (2006) reached five basic conclusions. First, interdisciplinary rehabilitation is beneficial over spontaneous recovery. Second, rehabilitation has no impact on mortality although it does influence quality of life. Third, there is conflicting evidence over which therapies are beneficial and many appear to have little direct benefit other than giving the patients a goal and social interaction. Fourth, there is strong evidence that greater intensity of therapies is beneficial over the short run. Fifth, there is no evidence regarding the timing and duration of therapy. In fact, one of the clear problems related to timing and duration is the willingness of insurers, whether they are private or government-based, to fund extensive rehabilitation. We can add one additional conclusion, which is that even if studies appear to be beneficial there is little known about why. That is, the mechanism whereby the treatment affects plasticity is usually unknown. It is here that the animal studies may inform us.

The single most effective postinjury experience in brain-injured laboratory animals is complex housing (for a review, see Kolb and Whishaw, 1998). This type of experience has been successfully used to facilitate functional restitution from experimental brain damage of varying etiologies (e.g., Kolb and Elliott, 1987; Will and Kelche, 1992; Johansson, 1996; Biernaskie and Corbett, 2001). The nature of the complex housing varies but the key features appear to be a novel and changing environment, a lot of exercise, and social interaction. The effects of this type of experience can be seen even in gross measures such as brain weight as well as dendritic measures such as length and complexity. In addition, there are non-neuronal changes too such as increases astrocyte number and complexity, and even in angiogenesis. The benefits of this experience is surprisingly general, including chronic improvements in both cognitive and motor functions.

Why is complex housing so beneficial? One clue comes from evidence of altered gene expression in the brain (Rampon et al., 2000). Changes in gene expression may alter the production of a variety of proteins, such as those involved in the synthesis of neurotrophic factors known to facilitate synaptic plasticity (e.g., Johansson, 2000).

How does this translate to humans? One constraint is on duration of the therapy: it is impractical to implement treatments that are 24 h/day for humans. This duration is likely not necessary in the animal studies, but it is convenient experimentally. Nonetheless, an effective treatment for humans would have to be intense, daily, and interdisciplinary including cognitive, social, and physical therapy. A useful laboratory animal experiment would be to titrate the amount of complex housing, perhaps comparing the effectiveness of 2, 4, 6, and 24 h/day.

A second type of effective experience in changing neural circuitry is tactile stimulation (e.g., Richards et al., 2012). Both adult and infant rats benefit from tactile stimulation using light touch with a fine brush several times daily for 15 min for 2–3 weeks after the brain injury. Both adult and infant rats with motor cortex or prefrontal injuries show marked functional improvements (e.g., Gibb et al., 2010; Kolb and Gibb, 2010). This is correlated with changes in dendritric length or spine density in cortex adjacent to the injuries. As shown in Figure 3, the rats with infant lesions showed a reversal of the decrease in spine density related to the injury. In contrast, rats with adult lesions, who showed extensive atrophy of adjacent cortical pyramidal neurons, showed an increase in dendritic length, not shown by shams, related to the tactile stimulation.

Recently, Livingston-Thomas et al. (2013, 2014) devised a novel behavioral approach, which they called voluntary forced use movement therapy. Rats were placed in plastic pet activity balls for 30 min per day for 21 days beginning 5 days after ischemic stroke. The therapy resulted in small but consistent acceleration of forelimb performance in several behavioral tests. The functional improvement was associated with an increase in migrating neural precursor cells originating in the subventricular zone as well as increased expression of BDNF (brain-derived neurotrophic factor) that were presumed to be in microglia.

Few laboratory studies have examined the beneficial effects of physical therapy on recovery. One emerging strategy is to implement specialized training protocols, with the best known being constraint-induced movement therapy for the arm and hand (Wolf et al., 2002). More recently robotic devices (e.g., Hidler et al., 2009) behavioral shaping, bilateral arm training (Lin et al., 2010), body weight-supported treadmill training (Dobkin et al., 2006; Duncan et al., 2007),task oriented physical therapy (Jonsdottir et al., 2010), and music therapy (Schneider et al., 2007) have also proven effective. The reasons for the effectiveness of these treatments is unknown but they presumably lead to synaptic changes that may be identified in mapping studies using noninvasive imaging or intracortical transcortical magnetic stimulation (TMS). Indeed, a recent study by Amengual et al. (2013) used music-supported therapy followed by TMS and found improved motor functions correlated with plastic changes in the form of increased cortical excitability following the training.

### Pharmacotherapy

We showed above that many drugs are extremely effective in stimulating neuroplastic changes in cerebral neurons, and especially psychomotor stimulants. Early studies by Feeney and colleagues (e.g., Feeney and Sutton, 1987) used amphetamine as a postinjury treatment following unilateral motor cortex injury and found striking benefits provided that the animals received physical therapy while under the influence of the drug (see also Goldstein, 2003). One complication of the amphetamine treatment is that lesion size, location, and route of administration appear to influence efficacy. Moroz and Kolb (2005) compared the effect of amphetamine on focal and extensive unilateral ischemic injuries finding that whereas amphetamine was useful in the former condition, it was not helpful with the larger injuries. This may explain why clinical studies with amphetamine have had mixed success. Clinical studies tend to select patients most in need of therapy, which are those with larger injuries. (Alaverdashvili et al., 2007) made small lesions similar to those of Moroz and Kolb but administered the same dose (1 mg/kg) orally rather than subcutaneously. They found no beneficial effect of the drug.

In unpublished studies we used amphetamine after bilateral medial frontal lesions, finding no benefit on the performance of cognitive tests. Given that all of the positive preclinical studies using amphetamine have been with animals with motor cortex injuries, the jury is still out on whether it will be effective for cognitive improvement. As mentioned above, it is usually necessary to make bilateral injuries in rodents to obtain robust cognitive deficits. Thus, it may not be the motor/cognitive distinction that is important but rather the unilateral/bilateral difference.

**Figure 4:**
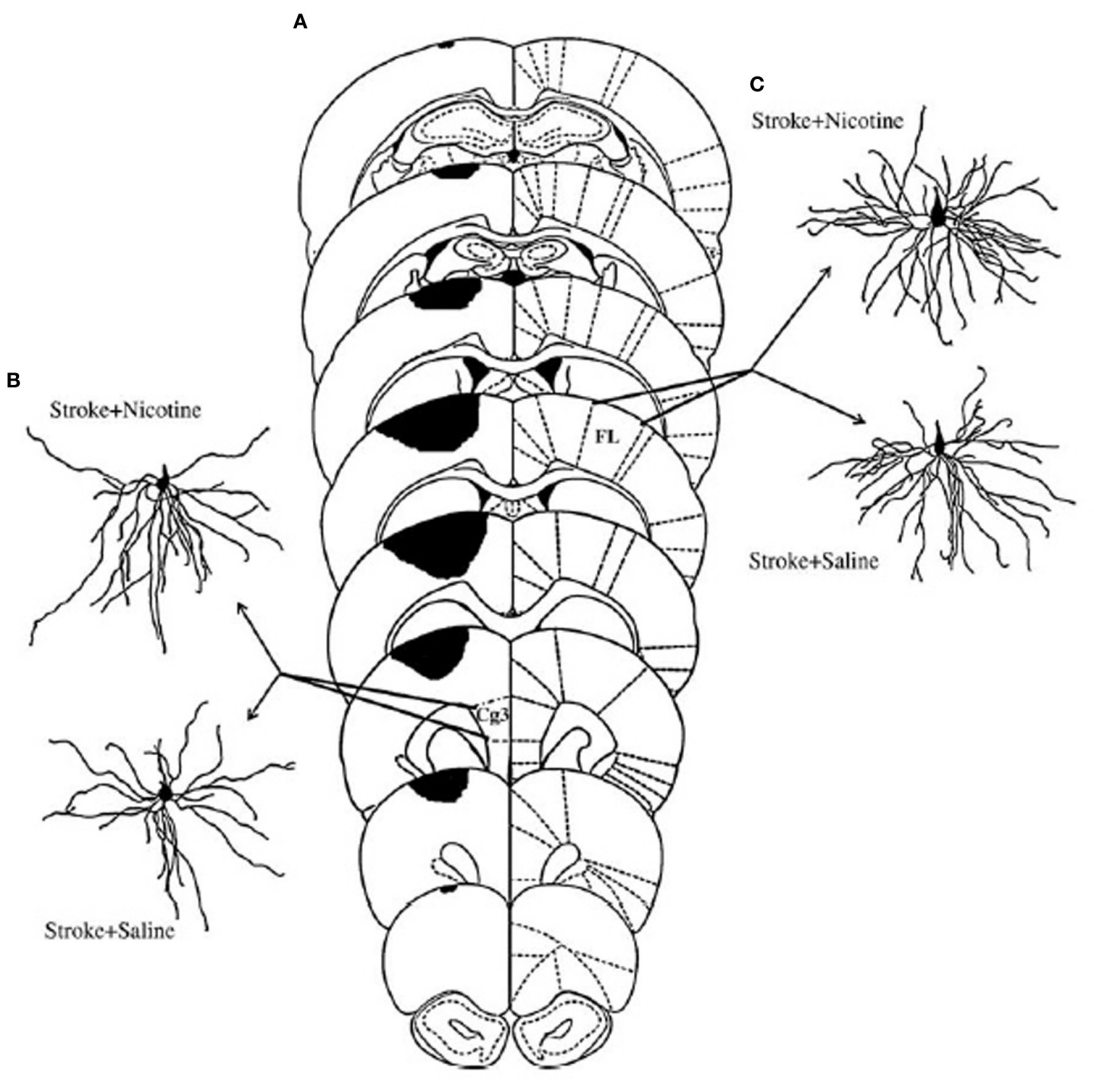
**(A)** Extent of ischemic injury across 8 planes of measurement. **(C)** Illustrate representative camera lucida drawings of layer V pyramidal neurons in anterior cingulate cortex (Cg3) ipsilateral to the lesion **(B)**, or forelimb area (FL) in the contralesional hemisphere **(C)** from animals treated either with saline or nicotine. Increased dendritic length in the nicotine-treated animals was correlated with improved functional outcome (After Gonzalez et al., 2006).

Nicotine has also proven effective in stimulating postinjury functional improvement after ischemic motor cortex injury (Gonzalez et al., 2006). A low dose of nicotine was given subcutaneously twice daily for 12 days following the ischemia. Functional recovery was followed on several behavioral tasks for 7 weeks. All behaviors showed a benefit from the treatment, which was correlated with synaptic changes both ipsilateral and contralateral to the ischemia (see Figure 4). Once again, route of administration may be important. Lim et al. (2009) found no benefit of nicotine on skilled reaching after similar strokes to Gonzalez et al but they administered the same dose (0.3 mg/kg) orally rather than subcutaneously.

**Figure 5:**
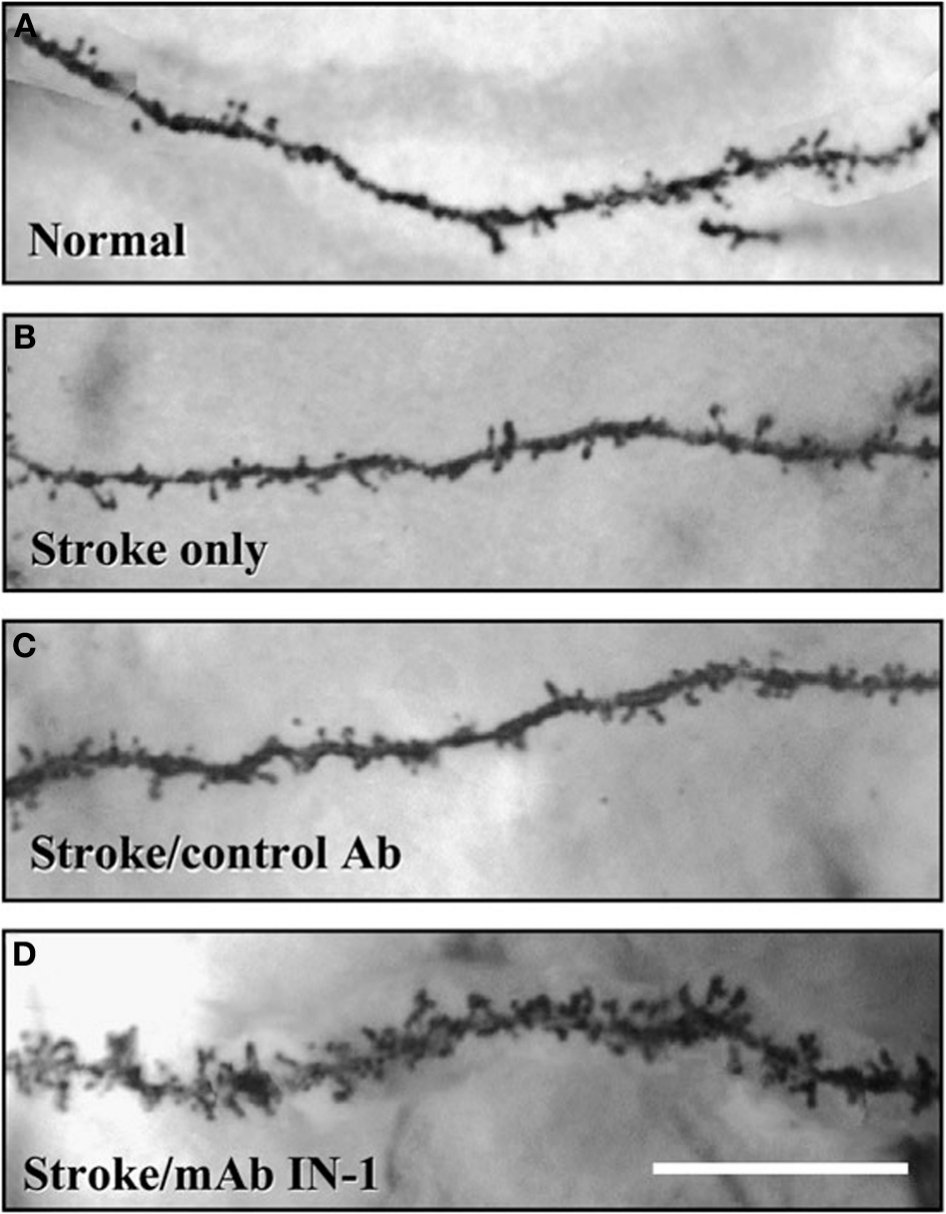
**Both dendritic length and spine density (shown here) in the contralateral forelimb cortex is enhanced by antibodies to NoGo-A (After** Papadopoulos et al., 2006).

Other types of pharmacotherapies have taken advantage of compounds that enhance axonal sprouting after cerebral injury. One example is an antibody to NoGo-A, a myelin-associated inhibiting axonal regeneration after central nervous system injury (for a review, see Kempf and Schwab, 2013). Papadopoulos et al. (2006) showed that administration of an antibody to NoGo-A stimulated axon generation and increased synaptogenesis in cortical pyramidal neurons (see Figure 5). These morphological changes were correlated with functional recovery on measures of skilled reaching. Hamadjida et al. (2012) performed a parallel study of motor cortex injury in monkeys who received NoGo-A. They found functional improvement in the treated animals along with sprouting and/or sparing of callosal projections to premotor cortex on the lesion side compared to untreated monkeys.

Because NoGo-A is an endogenous molecule found in oligodendrocytes and some neurons, it is reasonable to wonder if the expression of NoGo-A might be affected by other therapies. Zhao et al. (2013) treated rats with motor cortex ischemia with constraint-induced therapy for 3 weeks poststroke. After removal of the constraint, the animals were significantly improved on a beam-walking test and this was correlated with increased crossings of contralateral corticospinal axons to the denervated cervical spinal cord and a reduction in the expression of Nogo-A receptor in the peri-infarct cortex.

Another compound that has proven effective is inosine, a compound that regulates axon outgrowth through changes in gene expression. Several studies have shown that inosine stimulates the projection of new axons from the undamaged side of the brain to denervated areas of midbrain and spinal cord (Chen et al., 2002; Smith et al., 2007; Zai et al., 2009).

**Figure 6:**
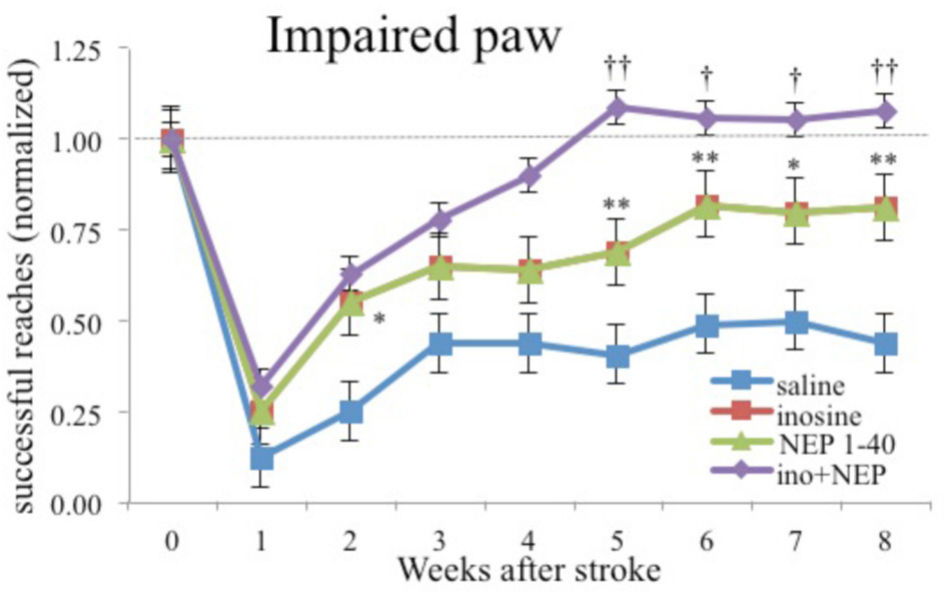
**Inosine combined with NoGo-A receptor blocker (NEP1-40) restores performance with the impaired paw to preoperative levels**. Similar results were found with a combination of inosine and complex housing (after Zai et al., 2011). ^*^ and ^**^ different from saline-treated controls significant at *p* < 0.05 and *p* < 0.01 respectively. ^†^ and ^††^ different from rats treated animals with NEP1-40 at *p* < 0.05 and *p* < 0.01 respectively.

Not only is inosine effective alone, it is synergistic in enhancing recovery of skilled reaching when given in combination with either antibodies to NoGo-A or complex housing (see Figure 6; Zai et al., 2011). The level of performance was one of the few examples of performance on a skilled task returning to preoperative levels following motor cortex injury plus treatment. Unfortunately, the authors did not do kinematic analysis so it is unclear how normal the actual movements were.

### Cell-based therapy

The possibility that mammalian brains can generate neurons after an injury can be traced to Altman (1962) but it was not until Reynolds and Weiss (1992) isolated and identified multipotent stem cells from the adult forebrain that more recent interest was spurred in the potential therapeutic value of stem cells. There are now many papers showing that following a stroke in rodents there is an enhanced proliferation within the SVZ followed by neuronal migration toward the ischemic regions (Arvidsson et al., 2002; Parent et al., 2002; Jin et al., 2003; Zhang et al., 2004). This spontaneous proliferation is not sufficient to restore functions, however. But what if it were possible to increase this spontaneous proliferation in some way? There appear to be two primary ways: have an early brain injury or infuse growth factors.

In the early 1990s we noticed that rats with medial frontal lesions around 7–12 days of age sometimes appeared to regenerate the lost tissue but this did not happen either before or after those ages. Following the paper of Reynolds and Weiss we hypothesized that perhaps the spontaneous regeneration was due to a redirection of newly generated neurons from the rostral migratory stream to the olfactory bulb into the region of injury (Kolb et al., 1998). This regrowth was associated with functional recovery. If the regeneration was prevented, the behavior did not recover (Kolb et al., 2012b) or if the tissue was removed in adulthood, the behavioral improvement was reversed (Dallison and Kolb, 2003). The timing of the critical period for this to happen correlated with the emergence of cells expressing a neurogrowth factor, Fibroblast Growth Factor-2 (Monfils et al., 2006). This led to the idea that adding a FGF-2 after an injury might stimulate the production of more neurons in brains that would not normally show this compensatory response, a possibility made even more likely by evidence that FGF-2 can stimulate proliferation of stem cells *in vitro*. Indeed, when rats with motor cortex lesions at postnatal day 10 received i.p. injections of FGF-2, it not only stimulated the proliferation of cells but these cells migrated to the site of injury, formed connections with the spinal cord, and supported apparently complete recovery of fine motor functions (e.g., Monfils et al., 2008). Unfortunately, this only appears to work in the developing brain and does not occur in adulthood, although the reason for this difference is not known.

Another powerful mitogen for adult neural stem cells is epidermal growth factor (EGF), either *in vitro* or *in vivo*. Intraventricular infusion of a combination of EGF and erythropoietin (EPO) following motor cortex strokes in adulthood and stimulates the growth of new neural precursor cells in the subventricular zone (Kolb et al., 2007). A plug of tissue containing both glia and immature neurons formed in the infarcted tissue. Cell tracking studies revealed that cells had migrated from the SVZ. Like the FGF-2 experiments, this was correlated with significant, but by no means complete, functional improvement on several motor tasks. In contrast to the FGF-2 studies there were also few direct connections into the original brain tissue. This led the authors to suggest that the plug of tissue was acting as some type of “factory” for chemicals that enhanced the function of the perilesional tissue. It is also possible that the EGF+EPO had modified existing neural circuits, which in turn led to enhanced behavioral outcome.

There are now many studies that have used a variety of growth factors to stimulate proliferation of neural stem cells after injury (for a review see Dibajnia and Morshead, 2013). Many of these factors have been shown to act directly on their respective receptors but they may also mediate proliferation of precursor cells but Dibajnia and Morshead point out that they may have indirect effects too via immunomodulation, neuroprotection, and angiogenesis.

There are significant challenges in moving to the clinic with compounds to increase the proliferation of neural precursor cells. Many of these compounds are powerful mitogens, leading to potential harmful effects. Another issue is the route of delivery of activation factors. Peripheral routes (intravenous, subcutaneous, intraperitoneal) may have widespread systemic effects and may not enter the brain in sufficient strength to produce the required number of neurons. Direct injection into brain tissue or ventricles is invasive and could lead to other complications. There is also the problem of white matter injury in larger brains that we mentioned earlier and whether the growth factors would affect white matter. In addition, there is the problem of the much greater distance that proliferating cells would have to migrate in humans vs. rodents. Finally, older brains have fewer neural precursor cells in the subventricular zone, likely making it more difficult to stimulate enough cells to make a difference. Clearly, transition to the clinic is some time away.

## Conclusions

A key property of the nervous system is the capacity to change after experience, including injury. As we understand the principles that control plasticity in the normal brain novel treatment strategies are being developed to stimulate recovery after cerebral injury in people.

A significant challenge in the coming decade is to devise ways to apply the knowledge generated in laboratory animal studies to develop successful rehabilitation strategies. Recent results of rodent studies are especially encouraging and could be implemented fairly easily. The first type of treatment to prove successful in animal studies was complex housing but several pharmacological treatments (antibodies to NoGo-A, inosine), and their combinations, show promise to surpass complex housing as the most effective type of treatment, however. Further combinations of rehabilitation strategies and other treatments may provide even better outcomes.

### Conflict of interest statement

The authors declare that the research was conducted in the absence of any commercial or financial relationships that could be construed as a potential conflict of interest.
